# Supplementary material for: Comparison of Twelve Ant Species and Their Susceptibility to Fungal Infection
Source: Insects. 2019 Aug 26;10(9):271. doi: 10.3390/insects10090271 (PMC6780858; doi:10.3390/insects10090271)
Supplement: Supplementary file 1 [file insects-10-00271-s001.pdf]

Table S1. Mean number of allogrooming events per recording of 30 ants.

	**Control**	***B. Bassiana***	***M. brunneum***
C. ligniperdus	0.08 ± 0.33	0.14 ± 0.34	0.12 ± 0.37
M. schencki	0.00 ± 0.00	0.01 ± 0.12	0.00 ± 0.00
M. ruginodis	0.04 ± 0.20	0.04 ± 0.26	0.03 ± 0.17
F. lugubris	0.03 ± 0.17	0.04 ± 0.20	0.01 ± 0.12
F. exsecta	0.04 ± 0.20	0.01 ± 0.12	0.00 ± 0.00
F. fusca	0.00 ± 0.00	0.00 ± 0.00	0.01 ± 0.12
F. cinerea	0.27 ± 0.65	0.39 ± 0.78	0.52 ± 0.88
F. sanguinea	0.01 ± 0.12	0.04 ± 0.26	0.01 ± 0.12
F. pratensis	0.04 ± 0.20	0.03 ± 0.17	0.03 ± 0.17
F. truncorum	0.01 ± 0.12	0.00 ± 0.00	0.04 ± 0.20
L. platythorax	0.00 ± 0.00	0.01 ± 0.12	0.03 ± 0.16
L. flavus	0.07 ± 0.38	0.04 ± 0.26	0.01 ± 0.12

Table S2. Mean number of trophallaxis events per recording of 30 ants.

	**Control**	***B. Bassiana***	***M. brunneum***
C. ligniperdus	0.01 ± 0.12	0.12 ± 0.37	0.07 ± 0.25
M. schencki	0.00 ± 0.00	0.00 ± 0.00	0.00 ± 0.00
M. ruginodis	0.03 ± 0.17	0.01 ± 0.12	0.00 ± 0.00
F. lugubris	0.04 ± 0.20	0.04 ± 0.20	0.08 ± 0.28
F. exsecta	0.01 ± 0.12	0.07 ± 0.26	0.04 ± 0.20
F. fusca	0.00 ± 0.00	0.04 ± 0.20	0.04 ± 0.20
F. cinerea	0.11 ± 0.36	0.23 ± 0.48	0.25 ± 0.49
F. sanguinea	0.04 ± 0.20	0.06 ± 0.23	0.03 ± 0.17
F. pratensis	0.13 ± 0.34	0.20 ± 0.47	0.11 ± 0.40
F. truncorum	0.04 ± 0.20	0.09 ± 0.29	0.04 ± 0.20
L. platythorax	0.00 ± 0.00	0.01 ± 0.12	0.00 ± 0.00
L. flavus	0.03 ± 0.16	0.00 ± 0.00	0.01 ± 0.12
